# Research on Cattle Behavior Recognition and Multi-Object Tracking Algorithm Based on YOLO-BoT

**DOI:** 10.3390/ani14202993

**Published:** 2024-10-17

**Authors:** Lei Tong, Jiandong Fang, Xiuling Wang, Yudong Zhao

**Affiliations:** 1 College of Information Engineering, Inner Mongolia University of Technology, Hohhot 010080, China; tonglei1235@163.com (L.T.);; 2 Inner Mongolia Key Laboratory of Perceptive Technology and Intelligent Systems, Hohhot 010080, China; 3 Inner Mongolia Synergy Innovation Center of Perception Technology in Intelligent Agriculture and Animal Husbandry, Hohhot 010080, China

**Keywords:** cattle, behavior recognition, multi-object tracking, behavior change analysis, YOLOv8

## Abstract

In smart ranch management, cattle behavior recognition and tracking play a crucial role in evaluating animal welfare. To address the issues of missed and false detections caused by inter-cow occlusions and infrastructure obstructions in the barn environment, this paper proposes a multi-object tracking method called YOLO-BoT. Built upon YOLOv8, the method first integrates dynamic convolution (DyConv) to enable adaptive weight adjustments, enhancing detection accuracy in complex environments. The C2f-iRMB structure is then employed to improve feature extraction efficiency, ensuring the capture of essential features even under occlusions or lighting variations. Additionally, the Adown downsampling module is incorporated to strengthen multi-scale information fusion, and a dynamic head (DyHead) is used to improve the robustness of detection boxes, ensuring precise identification of rapidly changing target positions. To further enhance tracking performance, DIoU distance calculation, confidence-based bounding box reclassification, and a virtual trajectory update mechanism are introduced, ensuring accurate matching under occlusion and minimizing identity switches. Experimental results demonstrate that YOLO-BoT achieves a mean average precision (mAP) of 91.7% in cattle detection, with precision and recall increased by 4.4% and 1%, respectively. Moreover, the proposed method improves higher order tracking accuracy (HOTA), multi-object tracking accuracy (MOTA), multi-object tracking precision (MOTP), and IDF1 by 4.4%, 7%, 1.7%, and 4.3%, respectively, while reducing the identity switch rate (IDS) by 30.9%. The tracker operates in real-time at an average speed of 31.2 fps, significantly enhancing multi-object tracking performance in complex scenarios and providing strong support for long-term behavior analysis and contactless automated monitoring.

## Simple Summary

This study presents a deep learning-based multi-object tracking method to enhance the accuracy of cattle detection and tracking, thereby improving farming efficiency and health management. The proposed method mitigates detection errors and omissions in complex environments while reducing identity switching and trajectory prediction errors through algorithmic enhancements. Experimental results show that the method achieves superior accuracy in cattle detection and behavioral tracking, operating in real time at 31.2 frames per second. This approach provides strong technical support for long-term monitoring and contactless cattle management, offering considerable practical value.

## 1. Introduction

Effective livestock management is essential for cattle farms in the era of modern smart agriculture [1]. As these farms expand, managers encounter the challenge of ensuring sufficient attention and care for each individual cattle. With the growth in herd size, traditional manual supervision becomes progressively inefficient and untimely. Consequently, tracking and monitoring the health status and behavioral changes of each cattle are crucial [2]. Recently, multi-object tracking algorithms integrated with deep neural networks gained significant attention for their effectiveness in cattle behavior recognition and tracking. These technologies allow managers to monitor cattle behavior and health with high accuracy, serving as powerful tools to enhance farming practices. Real-time monitoring of cattle behavior is vital for improving farming efficiency [3,4].

Two primary strategies for multi-object tracking are detection-free tracking (DFT) and detection-based tracking (TBD) [5]. In DFT, objects are manually labeled in the initial frame, and tracking is subsequently performed based on the object’s or region’s features within the bounding box. Appearance detection in DFT is modeled using integral histograms [6], subspace learning [7], sparse representations [8], or instance learning [9]. However, DFT has limited effectiveness in addressing challenges such as rapid object movement, occlusion, lighting changes, and appearance transformations. In contrast, TBD involves detecting objects in each frame and matching these detections using data association methods. The performance of TBD relies on both the accuracy of detection and the effectiveness of tracking [10]. Currently, extensive research is being conducted both domestically and internationally in the field of cattle behavior recognition and tracking. Researchers typically use YOLO series models [11] for object detection to achieve accurate recognition of cattle behavior. For tracking, most scholars apply the multi-object tracking (MOT) algorithm to monitor cattle movement trajectories and assign identity numbers (identity IDS).

In the field of object detection, due to the distinct external features of cattle, such as body characteristics and markings, object detection technologies can easily and accurately identify individual cattle and pinpoint their locations. Yu et al. [12] improved the YOLOv5 model by adding a small object detection head, enabling the identification of individual cattle behaviors. However, since the dataset used primarily comes from well-lit environments, the model’s generalization capability under varying lighting conditions or low-light environments may be limited, potentially affecting its detection accuracy in real-world applications. Hu et al. [13] enhanced the YOLOv5 model by incorporating an attention mechanism, achieving an average accuracy of 91.8% when identifying behaviors such as standing, walking, and lying down in grazing sheep. Nevertheless, this method encounters difficulties in behavior detection when sheep are in close proximity or are partially occluded, resulting in reduced identification accuracy. Li et al. [14] improved the YOLOv8n model by expanding the receptive field and adding an attention mechanism, thereby enhancing its ability to capture contextual features. The enhanced model achieved an average accuracy of 93.6% in recognizing nine different behaviors of beef cattle. The strength of this study lies in its dataset, which covers a wide range of behaviors and complex scenarios, such as varying lighting conditions, dense and sparse populations, and small distant objects, reflecting the diversity of actual farming environments. Wei et al. [15] introduced a multi-scale attention mechanism to improve the YOLOv8 model, enabling real-time and efficient detection and identification of individual pigs’ movements and aggressive behaviors. Although the model’s adaptability and robustness in diverse scenarios were significantly improved, there may still be a decline in recognition accuracy under extreme conditions, such as dense occlusion or drastic lighting changes. In the aforementioned studies, most methods primarily focus on identifying individual livestock behaviors. However, in real-world farming environments, particularly in barns, identifying multiple cattle behaviors presents more complex challenges, such as occlusion between cattle or by facilities, lighting variations, and changes in target scale. Therefore, this study focuses on recognizing multiple cattle behaviors in complex farming environments, aiming to improve the accuracy and stability of models in practical applications.

In the field of object tracking, due to the relatively large size and slow movement of cattle, multi-object tracking algorithms can effectively trace the movement trajectories of cattle and assign each individual a unique identity. Zheng et al. [16] optimized the ByteTrack [17] tracking algorithm by improving the state parameters in the Kalman filter, enabling more precise matching of tracking boxes to dairy cattle and demonstrating good multi-object tracking performance in natural environments. However, although the study tested different lighting conditions, the variations in lighting were minimal, and the robustness of the algorithm in complex lighting environments remains inadequately verified. Zheng et al. [18] later proposed a buffer-based multi-object tracking method, showing high accuracy and real-time performance in cattle behavior identification and tracking, though the limited number of recognized behaviors and self-occlusion among cattle still reduced recognition accuracy, restricting the method’s applicability. Huang et al. [19] developed a mobile pig counting algorithm based on an improved YOLOv5 and DeepSORT [20], which was designed for detecting and counting pigs. However, its accuracy is constrained in large-scale pig herds and confined spaces, and the counting accuracy tends to be affected when the pig population is large. Zhang et al. [21] applied an improved YOLOv3 network combined with the DeepSORT algorithm to achieve multi-object tracking of beef cattle. Although this approach performed well in well-lit daytime conditions, its tracking accuracy in more complex environments remains to be further verified. Fuentes et al. [22] introduced a method combining regions of interest (ROI) with the YOLOv5 and DeepSORT algorithms to continuously monitor cattle behavior. However, this model is relatively complex and demands high computational resources, which may limit its practicality in real-world deployment, especially in environments with limited hardware performance or where long-term monitoring is required. Fu et al. [23] proposed a method for cattle behavior recognition and tracking in barn environments using an improved YOLOv8n and BoTSORT algorithm for multi-object tracking. While this method demonstrated high accuracy from specific data collection angles, its performance is heavily influenced by the collection position and angle, limiting its applicability. Despite progress in multi-object tracking of cattle, current research still faces challenges such as occlusion, uneven distribution, and identity switching in complex farming environments, leading to insufficient tracking accuracy and stability. As the number of cattle increases and environmental conditions change, the difficulty in accurately identifying and tracking them grows. This study aims to address the challenges of multi-object tracking in complex farming environments, enhancing the accuracy and stability of models in real-world applications.

In response to issues such as uneven cattle distribution, occlusion, significant target size variation, and frequent identity switching during tracking, which lead to decreased accuracy in complex barn environments, this study proposes a multi-object tracking algorithm, YOLO-BoT. The algorithm efficiently tracks multiple cattle within a farm using a single surveillance camera. It leverages the advanced YOLOv8 algorithm from the YOLO series and enhances it by replacing the up-sampling layer, modifying the feature extraction module, and adding a dynamic object detector to improve the accuracy of cattle behavior recognition. In terms of tracking, the algorithm redefines the confidence detection boxes and introduces DIoU distance calculations [24] to improve matching accuracy and reduce identity switching issues. Additionally, a virtual trajectory update mechanism is implemented to minimize prediction error accumulation during periods of target loss. By achieving stable multi-object tracking of cattle, the algorithm facilitates the statistical analysis of long-term cattle behavior changes, enabling the evaluation of cattle vitality. This provides technical support for non-contact automatic monitoring of cattle behavior. The remainder of this paper is structured as follows: Section 2 presents the materials and methods. Section 3 showcases the experiments and results, followed by a discussion of future research directions and scope. Section 4 concludes the study.

## 2. Materials and Methods

### 2.1. Materials

#### 2.1.1. Data Acquisition

**Figure 1:**
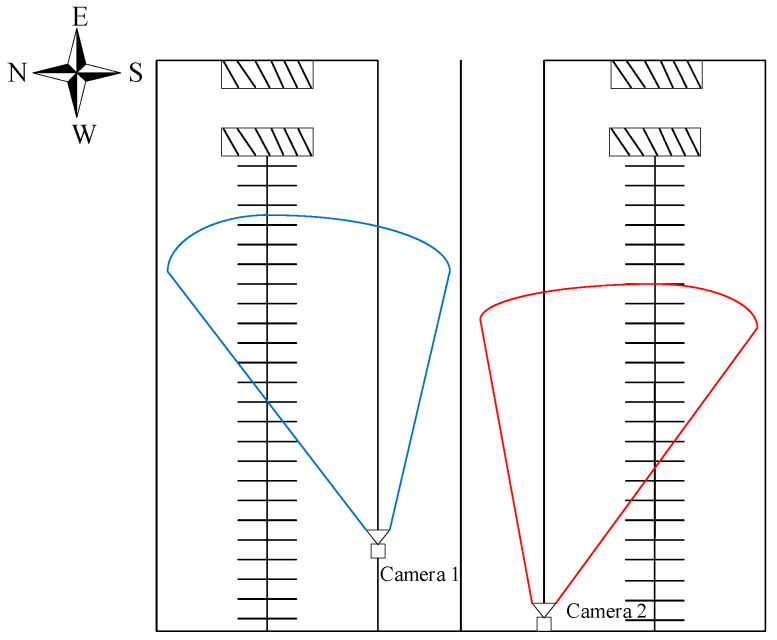
Schematic diagram of the cowshed. Camera 1, positioned near the entrance of the barn, is responsible for collecting behavioral data of the cattle in the blue area. Camera 2, located farther from the entrance, is responsible for collecting behavioral data of the cattle in the red area.

The cattle detection and tracking data used in this study were primarily collected from the Mengdelong Dairy Farm in Horinger County, Inner Mongolia, along with some publicly available datasets. The data collection methods included videos captured by surveillance cameras installed at the top of the barn, approximately 3 m above the ground, as well as videos recorded with handheld cameras and smartphones. After careful selection, videos with no visible targets or those affected by lens distortion were excluded, leaving 40 valid mp4 videos for analysis. These videos capture the activities of cattle at different times of the day, including both daytime and nighttime natural scenes. The video resolution is 1280 × 720 pixels, with a frame rate of 25 fps. To better illustrate cattle behavior, additional images of cattle activities were collected with a resolution of 4032 × 3024 pixels. The locations where the monitoring videos were recorded are shown in Figure 1.

**Figure 2:**
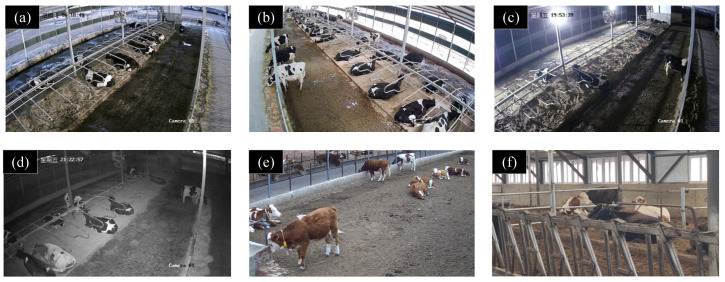
Examples of cattle data in different activity areas: (**a**) morning scene, (**b**) well-lit environment, (**c**) light interference, (**d**) night scene, (**e**) outdoor activity area, and (**f**) indoor activity area. The time in the top-left corner of the image represents the capture time of the data.

In this study, common cattle behaviors such as standing, walking, lying, feeding, and drinking were identified, with particular emphasis placed on less frequent behaviors such as crawling and fighting. An example of a video scene illustrating these behaviors is shown in Figure 2.

#### 2.1.2. Data Preprocessing

**Figure 3:**
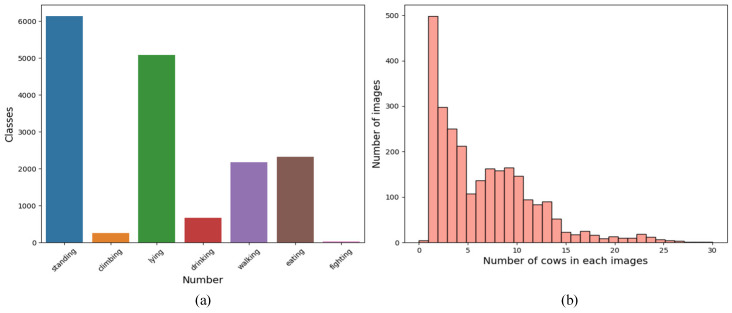
Analysis of the cattle behavior dataset: (**a**) analysis of cattle behavior labels, and (**b**) distribution of cattle count in each image.

Two datasets were established from the collected data: one for cattle behavior recognition models and the other for validating the effectiveness of the multi-object cattle tracking algorithm. Thirty of the videos were selected, and video frame extraction technology was used to save each frame as a jpg image. After removing redundant images, additional behavior-specific images captured by the camera were added to complement the frames extracted from the videos. After comprehensive screening, a total of 3484 images that met the requirements were obtained. These images were annotated for cattle behavior using the LabelImg 1.4.0 software, and the dataset was divided into training, validation, and testing sets in a 7:2:1 ratio. This resulted in 2440 images for the training set, 696 for the validation set, and 348 for the testing set. Figure 3a displays the number of labels for each behavioral category, totaling 16,673 targets. Standing and lying behaviors are the most frequent, while mounting and fighting behaviors are less common, reflecting the typical distribution of cattle behaviors in real farming environments. Figure 3b displays the distribution of the number of cattle per image in the dataset.

To evaluate the performance of the multi-object tracking algorithm, a multi-object tracking dataset was constructed and tested using 10 video clips, each 60 s long. These videos were labeled with serial numbers from 01 to 10, representing different scenes and time periods. The DarkLabel Ver.2.0 software was used to annotate the cattle tracking trajectories, generating a dataset compatible with the MOT17 tracking algorithm [25]. Each target’s ID, bounding box size, and coordinates were recorded and saved in txt format. Through manual observation, videos with a higher number of cattle and significant occlusion due to dense distribution were classified as dense videos, while videos with fewer cattle and less occlusion were categorized as sparse videos. Detailed classification is shown in Table 1, as referenced in [16]. Additionally, a re-identification (ReID) dataset was constructed using various perspectives and behaviors of 15 individual cattle, consisting of 16,288 images, with an average of 798 images per cattle.

### 2.2. Cattle Object Detection

#### 2.2.1. YOLOv8 Object Detection

For object detection, this study selects the YOLOv8 algorithm from the YOLO series as the base model, which includes five variants with different parameter sizes: n, s, m, l, and x. Among these, YOLOv8n is the model with the smallest number of parameters and the fastest detection speed. Considering that the model will eventually need to be deployed on edge devices with limited computing power, YOLOv8n was chosen as the base model, and further optimizations and improvements were made on this foundation.

#### 2.2.2. Feature Extraction C2f-iRMB Module

In complex cattle farming environments, the monitoring system must cover a wide range of areas, including both inside and outside the barn, as well as activity zones, all while meeting real-time requirements. As a result, the deployment of mobile devices becomes crucial. For cattle behavior recognition tasks, model lightweighting and accuracy improvement are key considerations. However, increasing the model’s parameters and complexity typically leads to higher computational resource demands.

**Figure 4:**
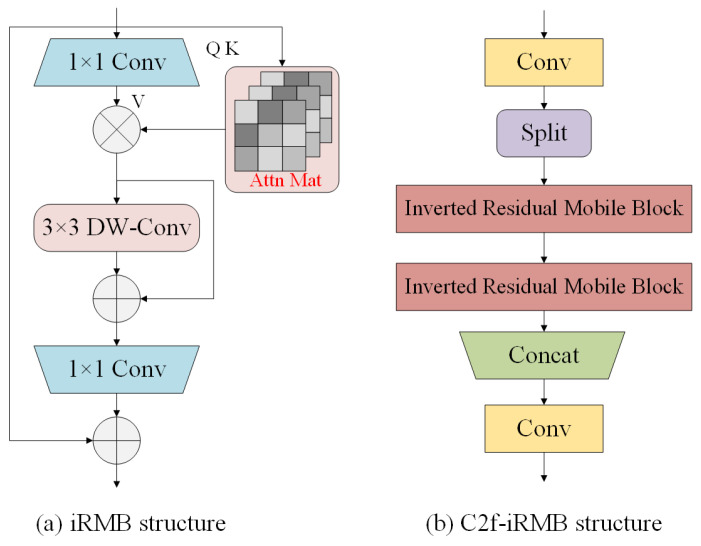
iRMB structure and C2f-iRMB structure.

To reduce redundant feature interference and lower model complexity, MobileNet introduced depthwise separable convolution, while MobileNetv2 further incorporated the inverted residual block (IRB) based on depthwise convolution, leading to a more lightweight model. However, these CNN models still face challenges in terms of accuracy. The inverted residual mobile block (iRMB) combines the lightweight characteristics of CNNs with the dynamic modeling capabilities of transformers, offering an efficient mobile network solution suitable for dense object detection tasks. Its structure is shown in Figure 4a [26]. iRMB optimizes computational efficiency and feature capture ability through the use of depthwise separable convolutions and a self-attention mechanism.

In this paper, the iRMB is integrated into the C2f module of the Neck component of the YOLOv8 network, resulting in the new C2f-iRMB module, as illustrated in Figure 4b. Compared to the original C2f module, the C2f-iRMB enhances feature extraction efficiency and accuracy while reducing computational and parameter complexity. This improvement significantly enhances the performance of cattle behavior detection in complex scenarios.

#### 2.2.3. ADown Downsampling

Downsampling plays a key role in deep learning models by reducing the spatial dimensions of feature maps while increasing their depth, allowing for the retention of important information, reducing computational cost and memory consumption, and expanding the model’s receptive field. In this study, we introduce the lightweight downsampling operation ADown from the latest YOLO series model, YOLOv9 [27]. The ADown module leverages the advantages of both average pooling and max pooling, processing images through different paths. This approach preserves essential spatial information and features while reducing the number of parameters and computational complexity, ultimately enhancing overall detection accuracy.

**Figure 5:**
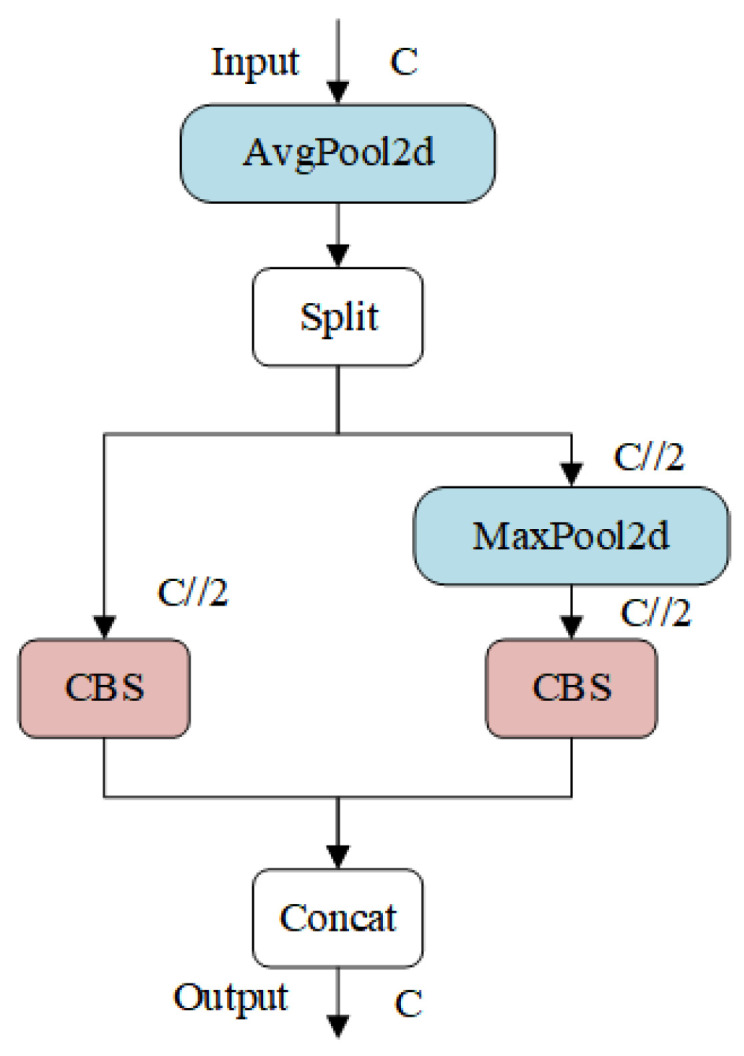
ADown downsampling structure.

The ADown module utilizes both average pooling and max pooling to capture diverse image features. Average pooling preserves background information, while max pooling highlights significant features, such as edges. The processed image is divided into two segments through a chunk operation, enabling independent processing of each segment and allowing the model to learn from multiple perspectives. One segment undergoes downsampling via a convolution with a stride of 2, which reduces dimensionality and extracts features. The other segment is subjected to max pooling followed by a 1 × 1 convolution to adjust channel numbers and retain crucial feature information. The results from these two segments are then combined to produce the final output. The structure of the ADown downsampling module is illustrated in Figure 5.

In this enhancement, the ADown downsampling module replaces the P3, P4, and P5 layers in the original backbone network, as well as the Conv convolution module in the Neck component. This modification not only improves detection accuracy, but also reduces the model’s computational workload.

#### 2.2.4. DyHead, a Dynamic Detection Head Based on Attention Mechanism

**Figure 6:**
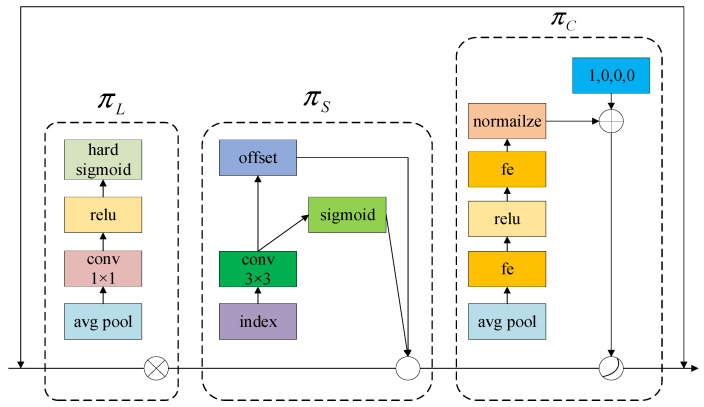
DyHead structure.

To address the limitations of single-scale prediction and the lack of dynamic learning capability in the original YOLOv8 detection head, this paper introduces the dynamic head (DyHead) module [28]. The DyHead module leverages a dynamic routing mechanism to enhance the fusion of contextual information, thereby improving multi-scale object detection performance. By dynamically adjusting the weights across different feature layers, DyHead effectively extracts multi-scale features, enhancing both detection accuracy and robustness. The DyHead module integrates scale-aware, spatial-aware, and task-aware attention through a self-attention mechanism. The formula for self-attention calculation is as follows:

(1) $$W\left(F\right)=\pi_{C}\left(\pi_{S}\left(\pi_{L}\left(F\right)\cdot F\right)\cdot F\right)\cdot F,$$

where W denotes the attention function, and F represents the input feature tensor $F\in R^{L\times S\times C}$. The symbols $\pi_{C}$, $\pi_{S}$, and $\pi_{L}$ correspond to the task-aware, spatial-aware, and scale-aware attention modules, respectively. These attention modules operate along the L, S, and C dimensions of the tensor. The structure of a single DyHead module is illustrated in Figure 6.

#### 2.2.5. Dynamic Convolution DyConv

In object detection tasks, traditional convolutional operations often struggle to adapt fixed convolutional kernels to objects of varying scales and positions, leading to feature redundancy and increased computational burden. To address these challenges, dynamic convolution (DyConv) introduces a novel feature extraction mechanism [29]. The core idea of DyConv is to dynamically adjust the sampling points of the convolution kernel through learnable offsets and attention mechanisms. This approach enhances both the accuracy and flexibility of feature extraction by adapting the convolution kernel’s position to the spatial structure of the input feature map. Consequently, it reduces redundant computations and improves overall model performance.

**Figure 7:**
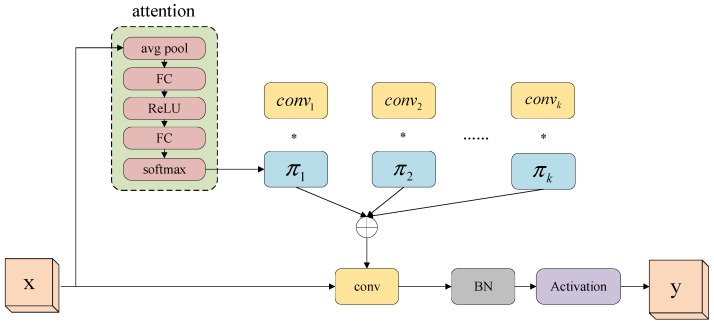
Dynamic convolution. The “*” represents element-wise multiplication of each convolution output with its attention weight.

The dynamic convolution layer functions similarly to a special dynamic perceptron with K kernels, as illustrated in Figure 7. Following this dynamic convolutional layer, batch normalization and activation functions are utilized to accelerate model training and enhance training accuracy. Additionally, a lightweight squeeze-and-excitation (SE) module is introduced to extract attention weights $\pi_{k}$. Unlike SENet, which provides attention mechanisms to channels, dynamic convolution assigns attention mechanisms to the convolution kernels. Through the SE module, the weight of each convolution kernel can be dynamically adjusted based on the input, generating an adaptive dynamic convolution.

**Figure 8:**
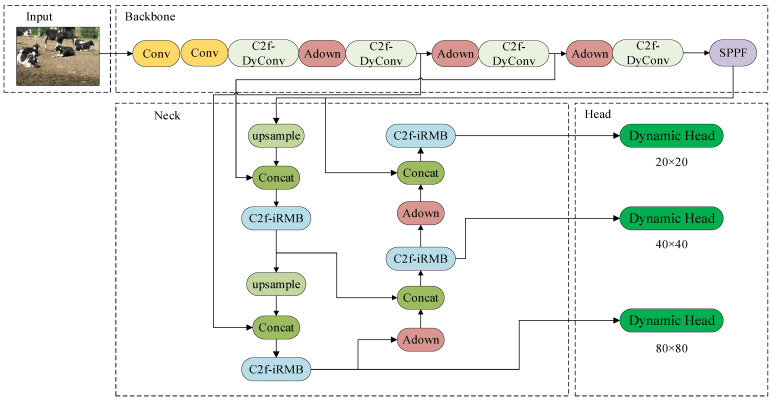
The improved YOLOv8n network architecture.

In this study, the C2f module in the Backbone network is enhanced by integrating the dynamic convolutional DyConv module, resulting in a new structure named C2f-DyConv. This integration replaces the original feature extraction module. The C2f-DyConv structure dynamically adjusts the weights of the convolution kernels based on the input data, enabling it to better adapt to features of varying scales, orientations, and shapes. This improvement significantly enhances the model’s flexibility and adaptability. The modified YOLOv8n structure is illustrated in Figure 8.

#### 2.2.6. Object Detection Evaluation Metrics

To assess the performance and effectiveness of cattle behavior recognition, this study employs several evaluation metrics: precision (P), recall (R), average precision (mAP@0.5), F1 score, number of model parameters (Params), model size, frame rate (FPS), and floating-point operations (GFLOPs). Model size refers to the final size of the saved model after training. A threshold of intersection over union (IoU) ≥ 0.5 is set to determine correct identification of cattle. The definitions of these metrics are provided by Equations (2)–(5).

(2) $$P=\frac{TP}{TP+FP}$$

(3) $$R=\frac{TP}{TP+FN}$$

(4) $$mAP=\frac{\sum_{c=1}^{C}AP\left(c\right)}{C}$$

(5) $$F1=\frac{2PR}{P+R}$$

Among them, true positives (*TP*) refers to the number of correctly identified positive samples; false negatives (*FN*) denotes positive samples that were incorrectly predicted as negative by the model; and false positives (*FP*) represents negative samples incorrectly classified as positive by the model. The mean average precision (mAP) is calculated as the average of the average precision (*AP*) values for detecting cattle behavior. In this study, *C* represents the number of detection categories, which is 7.

### 2.3. Cattle Multi-Object Tracking

#### 2.3.1. Multi-Object Tracking Algorithm for Cattle

In the field of multi-object tracking (MOT), data association is a crucial factor in determining tracking accuracy and stability. Existing MOT methods adopt various strategies to handle challenges in complex scenarios, such as dealing with targets that have similar appearances and irregular motion trajectories. For instance, DeepSORT utilizes position prediction and appearance features for two-stage data association; StrongSORT [30] integrates multi-layer features for weighted data association; ByteTrack first matches high-confidence detection boxes, followed by low-confidence ones to reduce missed detections; C-BIoU [31] employs a cascade matching approach, using a small buffer to match tracks with detections first, and then a larger buffer to associate unmatched tracks and detections; and OCSORT [32] introduces an observation-centric re-update (ORU) mechanism that leverages virtual trajectories to perform retrospective updates when a target temporarily loses association, correcting trajectory deviations to ensure continuity in complex environments. Although these methods each have their strengths, they still face issues such as false detections and missed detections in handling detection boxes with imbalanced confidence levels in complex environments. To address these challenges, this study improves the BoTSORT algorithm by redefining confidence thresholds, introducing DIoU distance calculations, and incorporating a virtual trajectory update mechanism. These enhancements aim to better handle the challenges of multi-object tracking in cattle, improving overall tracking accuracy and robustness.

**Figure 9:**
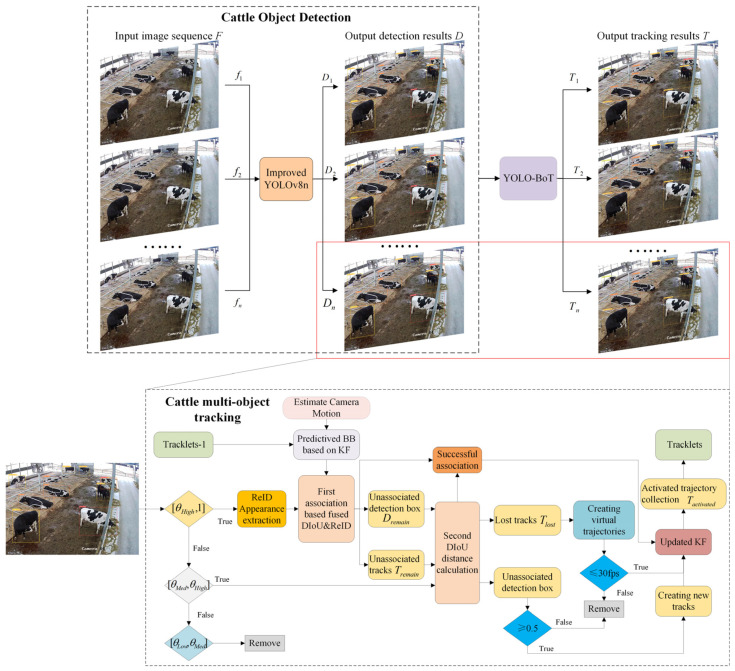
Flowchart for multi-object tracking of cattle.

In this study, the BoTSORT algorithm for multi-object tracking of cattle was enhanced, and the tracking process is illustrated in Figure 9. Initially, detection results from the YOLOv8n model were classified into high $\left[\theta_{High},1\right]$, medium $\left[\theta_{Med},\theta_{High}\right]$, and low $\left[\theta_{Low},\theta_{Med}\right]$ confidence categories, with low-confidence detections being discarded. Subsequently, the Kalman filter was used to predict the position of the trajectory set $T_{t-1}$ in the previous frame, and the predicted bounding box position was adjusted using camera motion compensation (CMC). Object appearance features were extracted and associated with detection frames using the ReID model. The matching process involved two rounds of matching mechanisms. In the first round, DIoU distance and appearance feature distance were employed for matching. Successfully matched trajectories updated their states and were added to the activated trajectory collection $T_{activated}$. Unmatched trajectories and detected frames were added to the trajectory retention collection $T_{remain}$ and detected frames retention collection $D_{remain}$, respectively. In the second round, high-score detection frames from $D_{remain}$ were used for DIoU distance calculations and matched with trajectories in $T_{remain}$. Successfully matched trajectories were updated and added to $T_{activated}$, while unmatched trajectories were moved to $T_{lost}$. For unmatched high-score detection frames, if the confidence exceeded the threshold, a new trajectory was created and added to $T_{activated}$; otherwise, unmatched trajectories were added to $T_{lost}$. Remaining detection frames that did not match in the second round were discarded. To manage unmatched trajectories, a virtual trajectory update mechanism was introduced for trajectories in $T_{lost}$. The Kalman filter parameters were re-updated and matched according to virtual trajectories, with a threshold set to delete trajectories that remained unmatched beyond 30 frames. Finally, the Kalman filter was used to predict the next frame positions of trajectories in $T_{activated}$, resulting in $T_{t}$. By reclassifying confidence levels, using DIoU distance, and incorporating the virtual trajectory updating mechanism, the method effectively reduces misdetection interference, enhances matching accuracy, ensures trajectory continuity and stability, and thus improves overall detection accuracy and tracking performance. In the bull multi-object tracking experiments, the threshold $\theta_{High}$ was set at 0.5 for high-score detection frames and $\theta_{Med}$ at 0.3 for medium-score detection frames.

#### 2.3.2. Improved Tracking Algorithm

The original BoTSORT algorithm introduces a matching method that integrates motion and appearance information to enhance tracking performance. This method employs an IoU + ReID fusion mechanism for matching. Specifically, IoU is used to quantify the overlap between the detection frame and the predicted trajectory, facilitating the matching process. The IoU is calculated as follows:

(6) $$IoU=\frac{\;\mathrm{Area}\;\mathrm{of}\;\mathrm{Intersection}}{\mathrm{Area}\;\mathrm{of}\;\mathrm{Union}}.$$

The intersection over union (IoU) is calculated as the ratio of the area of intersection to the area of union between two bounding boxes. Specifically, the area of intersection refers to the region where the two bounding boxes overlap, while the area of union is the total area covered by both bounding boxes combined. The IoU value ranges from 0 to 1, with a higher value indicating greater similarity between the bounding boxes. To enhance the accuracy of the matching process, this study introduces the distance intersection over union (DIoU) as an alternative. DIoU extends IoU by considering not only the overlapping area, but also the distance between the centers of the two bounding boxes. The formula for DIoU is as follows:

(7) $$DIoU=IoU-\frac{\rho^{2}\left(b,b^{gt}\right)}{C^{2}}.$$

In the distance intersection over union (DIoU) calculation, b and $b^{gt}$ represent the coordinates of the centroids of the predicted and ground truth bounding boxes, respectively. The term $\rho^{2}\left(b,b^{gt}\right)$ denotes the squared Euclidean distance between these centroids, and *CC* represents the diagonal length of the smallest enclosing rectangle that encompasses both bounding boxes. By incorporating DIoU in place of IoU for matching calculations, the method more accurately measures object similarity by considering both the overlap of the bounding boxes and the distance between their centers. This approach significantly enhances matching accuracy, reduces identity switching, and improves overall tracking performance.

**Figure 10:**
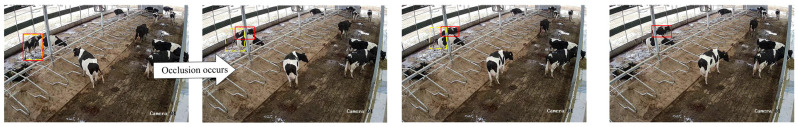
Schematic representation of the tracking process leading to object loss due to occlusion: The red solid line denotes the detection frame, while the yellow dashed line represents the predicted frame.

To enhance the reliability of the trajectory state, trajectory management updates both the prediction and correction steps of the Kalman filter for each frame to ensure state accuracy. The trajectory state is updated as described in Equation (8). The transformation matrix is denoted as $H_{t}$, and the Kalman gain is represented by $K_{t}$ (Equation (9)). The updated trajectory state covariance matrix $P_{t|t-1}$ is computed by predicting the trajectory state covariance matrix $P_{t|t-1}$ using $K_{t}$ and $H_{t}$, as shown in Equation (10).

(8) $${\hat{x}}_{t|t}={\hat{x}}_{t|t-1}+K_{t}(z_{t}-H_{t}{\hat{x}}_{t|t-1})$$

(9) $$K_{t}=P_{t|t-1}H_{t}^{T}{\left(H_{t}P_{t|t-1}H_{t}^{T}+R_{t}\right)}^{-1}$$

(10) $$P_{t|t}=P_{t|t-1}-P_{t|t-1}K_{t}H_{t},$$

where $R_{t}$ denotes the detection noise covariance matrix; ${\hat{x}}_{t|t}$ represents the trajectory state vector; and $P_{t|t}$ is the trajectory state covariance matrix. When the trajectory is lost, the Kalman filter is unable to update the trajectory state, resulting in the accumulation of prediction errors over time, which can lead to target loss. The schematic diagram illustrating target loss due to the accumulation of prediction errors is shown in Figure 10.

In Figure 10, the red solid line represents the detection frame, while the yellow dashed line indicates the prediction frame. The object is occluded from 06:58:00 to 06:58:03 and is successfully tracked at 06:58:03 when the prediction frame aligns with the detection frame. However, at 06:58:04, the prediction frame fails to match the detection frame due to its inability to update to the correct object position quickly enough, resulting in object loss. To address this issue, this paper introduces a virtual trajectory update mechanism. This mechanism combines the detection state vector prior to object loss with the state vector after re-detection to generate virtual observations, thereby updating the Kalman filter state during object loss to minimize prediction errors.

To ensure the continuity and accuracy of the trajectory during brief object losses, this study introduces cubic spline interpolation to re-update the trajectory [33,34]. Specifically, a cubic spline interpolation function is constructed using detection results from several frames before and after the loss. The detection state vectors, denoted as $z_{i}={\left[x_{i},y_{i},w_{i},h_{i}\right]}^{T}$, represent the position and size information of the object in the i-th frame. These state vectors are used to construct the cubic spline interpolation function $S_{t}(t)$.

(11) $$S_{t}(t)=a_{i}+b_{i}t+c_{i}t^{2}+d_{i}t^{3},$$

where $a_{i}$, $b_{i}$, $c_{i}$, and $d_{i}$ are the interpolation coefficients determined by the cubic spline interpolation method. The coefficients are computed to satisfy the following conditions: the interpolation function must match the state vector $z_{i}$ at the given detection point $t_{i}$, and the function must have continuous first and second derivatives at the boundaries between intervals. These conditions are expressed by the following system of equations:

(12) $$\left\{\begin{array}{l} S_{i}(t_{i})=z_{i}\\ S_{i}(t_{i+1})=z_{i+1}\\ S_{i}^{\prime }(t_{i+1})=S_{i+1}^{\prime }(t_{i+1})\\ S_{i}^{\prime \prime }(t_{i+1})=S_{i+1}^{\prime \prime }(t_{i+1}) \end{array}\right..$$

The time interval is defined as:

(13) $$\Delta t=t_{i+1}-t_{i}.$$

By solving the above equations, the cubic spline interpolation function $S_{t}(t)$ can be derived for each interval, allowing for the calculation of virtual observations $z_{t}^{\prime }$. Specifically, for a time point $t_{k}$ where the object is missing, its virtual observation can be determined using the interpolation function $S\left(t_{k}\right)$:

(14) $$z_{t}^{\prime }=S\left(t_{k}\right).$$

The state vector is updated using the generated virtual observations $z_{t}^{\prime }$, as indicated in Equation (15):

(15) $${\hat{x}}_{t|t}={\hat{x}}_{t|t-1}+K_{t}(z_{t}^{\prime }-H_{t}{\hat{x}}_{t|t-1}).$$

#### 2.3.3. Multi-Object Tracking Evaluation Metrics

To evaluate the performance of cattle tracking algorithms, the following metrics are employed: high order tracking accuracy (HOTA), multiple object tracking accuracy (MOTA), multiple object tracking precision (MOTP), mostly tracked ratio (MTR), mostly lost ratio (MLR), identity switches (IDS), and frames per second (FPS). Higher values of MOTA, MOTP, HOTA, IDF1, and MTR, as well as a higher FPS, indicate better tracking performance. Conversely, lower values of MLR and IDS suggest improved performance [23].

HOTA introduces a higher-dimensional tracking accuracy metric, providing a more comprehensive assessment of multi-object tracker performance. It is calculated as shown in Equation (16).

(16) $$HOTA=\sqrt{DetA\cdot AssA}=\sqrt{\frac{\sum_{C\in TP}A(c)}{TP+FN+FP}}$$

(17) $$A(c)=\frac{TPA(c)}{TPA(c)+FPA(c)+FNA(c)},$$

where DetA denotes the detection accuracy score and AssA denotes the association accuracy score. TP refers to the number of positive samples, FN refers to the number of the positive samples predicted by the model to be negative, and FP denotes the number of negative samples where the model incorrectly predicts a positive result. For a given point C that belongs to TP, the corresponding ground truth trajectory can always be determined. A(c) represents the association accuracy, as defined by Equation (17). Here, TPA(c) denotes the accuracy of correct associations, FPA(c) represents the accuracy of predicted trajectories with no or incorrect associations, and FNA(c) denotes the accuracy of predicted trajectories with no or incorrect associations due to association errors.

Multiple object tracking accuracy (MOTA) measures the occurrences of object misdetection, omission, and identity switching throughout the tracking process. It is calculated as shown in Equation (18):

(18) $$MOTA=1-\frac{\sum_{t}\left(FN+FP+IDS\right)}{\sum_{t}GT}.$$

In Equation (17), FN represents the number of objects that the tracker fails to recognize and track, FP denotes the number of objects that the tracker misidentifies, IDS indicates the number of times the object ID switches during tracking, and GT refers to the total number of objects labeled in the dataset.

MOTP measures the accuracy of the predicted object frames relative to the ground truth labels throughout the tracking process. It is calculated as shown in Equation (19):

(19) $$MOTP=\sum_{t,i}\frac{d_{t,i}}{\sum_{t}c_{t}},$$

where $d_{i,t}$ represents the distance between the given object and its paired hypothetical position in frame t; $c_{t}$ represents the number of matches between objects and hypothesis positions in frame t, and i represents the current detection object.

IDF1 represents the ratio of correctly recognized detections to the average number of true and computed detections. A higher IDF1 value indicates that the algorithm is more stable in accurately tracking a specific object.

(20) $$IDF1=\frac{2IDTP}{2IDTP+IDFP+IDFN}$$

where IDTP denotes the number of objects correctly recognized and tracked by the tracker, IDFP represents the total number of objects incorrectly tracked despite having the same ID, and IDFN indicates the total number of objects that were lost in tracking while their IDS remained unchanged.

## 3. Experimental Results

### 3.1. Experimental Platform and Parameter Settings

The experiments were conducted on a Windows 10 operating system with an Intel^®^ Core™ i7-10700F CPU running at 2.90 GHz, 64 GB of RAM, and an NVIDIA GeForce RTX 4070 graphics card. The algorithm development was performed using Visual Studio Code, with Python 3.9 as the programming language and PyTorch 1.10.0 as the deep learning framework.

To assess the performance of the proposed YOLO-BoT algorithm for cattle object tracking in complex farming environments, two experiments were conducted: (1) cattle object detection experiments to evaluate the effectiveness of the improved YOLOv8n model, and (2) cattle multi-object tracking experiments to analyze the performance of the enhanced BoTSORT algorithm. For the cattle object detection experiments, the model was trained using a custom cattle behavior dataset. The training parameters were set as follows: a batch size of 16, 200 training epochs, a learning rate of 0.01, and the SGD optimizer. GPU acceleration was enabled by default. For the SORT tracking algorithm, the IoU threshold was set to 0.7, the confidence threshold to 0.5, and all other parameters were left at their default values.

### 3.2. Analysis of Cattle Testing Results and Accuracy Evaluation

#### 3.2.1. Cross-Validation Experiments

To evaluate the robustness and generalization ability of the model, this study employed 5-fold cross-validation. Specifically, the training and validation sets were combined into a single dataset (a total of 3136 images), which was then divided into five subsets. In each experiment, four of these subsets were used as the training set, while the remaining one served as the validation set. This process was repeated five times to ensure that each subset participated in the validation phase. This approach helps minimize evaluation bias caused by uneven data partitioning, providing a more reliable assessment of the model’s performance.

In each fold of the experiment, the model underwent the standard training process and was then evaluated on the validation set. Performance metrics such as precision, recall, F1 score, and mean average precision (mAP) were recorded. After completing all the folds, the average values and standard deviations of these metrics were calculated to provide a more stable assessment of the model’s performance. The results of these experiments are shown in Table 2.

As shown in Table 2, the model’s performance on the validation set was relatively consistent, with an mAP of 92.3% and a standard deviation of 0.9%, indicating minimal fluctuation across different folds and demonstrating good robustness. The standard deviations of precision (P) and recall (R) were 1.7% and 1.9%, respectively, while the F1 score had a standard deviation of only 0.3%, further confirming the model’s balance and consistency across folds. Based on the cross-validation results, the model with the highest mAP on the validation set was selected for the final evaluation on the test set. Table 3 presents the comparison of the selected best-performing model on the validation set with its results on the test set.

As shown in Table 3, the model that performed best on the validation set exhibited a slight decrease in performance on the test set. Specifically, the mAP dropped from 93.4% to 91.7%, and the F1 score declined from 89.2% to 87.7%. However, precision (P) increased from 89.6% to 90.9%. Although the F1 score decreased, the improvement in precision indicates that the model maintains good generalization ability on unseen data. This suggests that while the model may exhibit slight variations in its balance between precision and recall, it remains robust when applied to new, untested scenarios.

The recall rate on the test set experienced a more noticeable decrease, dropping from 88.9% to 84.7%, which may suggest an increase in missed detections in certain scenarios. However, the overall performance remains close to that observed on the validation set, indicating that the model can maintain high detection accuracy and robustness even when faced with more challenging environments. This suggests that the model has good potential for practical application, despite the slight increase in missed detections in more complex situations.

#### 3.2.2. Comparison Experiments of Different Models

To evaluate the effectiveness of cattle behavior recognition, the improved YOLOv8n model was compared with several other models, including YOLOv3-tiny, YOLOv5n, YOLOv6n, YOLOv7-tiny, RTDETR-r18, YOLOv9t, YOLOv10n, and the original YOLOv8n. The comparison focused on various metrics, such as precision, recall, mean average precision (mAP), frame rate, number of model parameters, floating-point operations (FLOPs), and model size. The performance of the models was evaluated using the test set data, and the comparison results are shown in Table 4.

As shown in Table 4, the improved YOLOv8n achieved an mAP of 91.7%, surpassing the other eight models, and improving by 1.5% compared to the original YOLOv8n. The precision (P) also increased from the original 86.5% to 90.9%, outperforming all other models, demonstrating significant progress in reducing false detections. In terms of recall (R), the improved YOLOv8n achieved 84.7%, which, while slightly lower than YOLOv6n, YOLOv7-tiny, RTDETR-r18, and YOLOv9t, still exceeded the original YOLOv8n and other models, indicating it maintained solid performance in controlling missed detections. Regarding FPS, the improved YOLOv8n retained good real-time performance, though slightly lower than the original YOLOv8n, YOLOv3-tiny, YOLOv5n, and YOLOv6n. It still outperformed YOLOv7-tiny, YOLOv9t, RTDETR-r18, and YOLOv10n, suggesting it remains competitive for real-world applications. In terms of model complexity, the improved YOLOv8n showed a moderate performance in parameters, GFLOPs, and model size, slightly higher than YOLOv5n and YOLOv9t, but significantly better than YOLOv6n, YOLOv7-tiny, and RTDETR-r18. This indicates that the enhanced model successfully maintains high detection accuracy while effectively controlling computational and storage costs, making it particularly suitable for deployment on edge devices with limited computing power. Overall, by incorporating inverted residual blocks, attention mechanisms, and dynamic convolution modules, the improved YOLOv8n achieved optimizations in both feature extraction and computational efficiency, proving its effectiveness in cattle behavior classification tasks. When comparing all nine models, the improved YOLOv8n demonstrated significant advantages in both performance and resource consumption.

In summary, the model proposed in this paper demonstrates certain advantages in accurately identifying cattle objects in complex farming environments.

#### 3.2.3. Network Improvement Ablation Experiments

**Figure 11:**
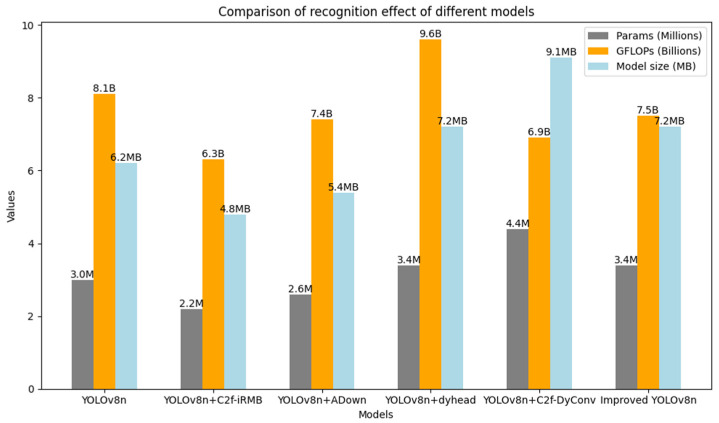
Ablation experiment results.

To further validate the effectiveness of the proposed improvements, ablation experiments were conducted on the methods described in Section 2.2.2, Section 2.2.3, Section 2.2.4 and Section 2.2.5. These experiments evaluated both the mean average precision (mAP) of overall cattle behavior detection and the average precision (AP) for different behavior categories, as detailed in Table 5. Additionally, to comprehensively assess the impact of the model enhancements, ablation experiments were performed on the number of model parameters, GFLOPs, and model size, with the results presented in Figure 11.

By analyzing Table 5 and Figure 11, the YOLOv8n + C2f-DyConv model demonstrated a 3.3% improvement in average precision for detecting cattle fighting behaviors compared to the original YOLOv8n. As shown in Figure 11, although the parameter count and model size increase slightly, the model maintains overall performance while providing higher accuracy for specific behavior categories and reducing computational costs, further enhancing its practical value in real-world deployment. When the C2f-iRMB module was incorporated into YOLOv8n, the average precision for identifying feeding behaviors increased by 7.6%. This improvement can be attributed to the C2f-iRMB module’s excellent feature extraction capability, which effectively captures the characteristics of cattle feeding behavior. Additionally, the parameter count, GFLOPs, and model size were reduced, further improving the model’s efficiency and practicality. With the introduction of the DyHead detection head, the recognition accuracy for all behaviors, except for feeding and lying down, improved significantly. DyHead proved particularly effective in handling behaviors with significant dynamics and posture changes, enhancing the model’s detection capabilities for these actions. After incorporating the ADown downsampling module, significant improvements were observed in the recognition of standing, walking, and fighting behaviors, with increases of 2%, 3.2%, and 3.3%, respectively. Moreover, the parameter count, GFLOPs, and model size were reduced. Compared to the C2f-iRMB module, the ADown module not only excelled in reducing parameter count and computational complexity, but also expanded the model’s receptive field, further improving its overall performance.

**Figure 12:**
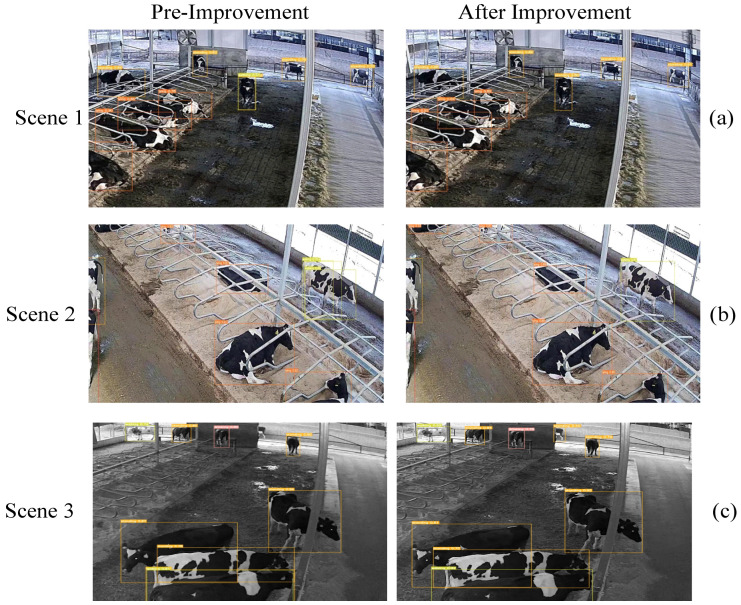
Comparison of algorithm improved cattle instance detection. In scenario 1, standing cattle are mistakenly detected as walking; in scenario 2, some behavioral features of lying cattle are missed and walking behavior is repeatedly detected; and in scenario 3, some features of walking behavior are missed.

The improved algorithm and the original YOLOv8n model were tested on cattle image instances, as shown in Figure 12. In Figure 12a, the YOLOv8n model incorrectly classifies the standing behavior of cattle positioned far from the camera as walking behavior. In Figure 12b,c, the YOLOv8n model fails to effectively capture and recognize the features of small objects, resulting in missed detections. In contrast, the improved model accurately identifies all cattle behaviors in both scenarios.

### 3.3. Experiments and Results Analysis of Multi-Object Tracking of Cattle

#### 3.3.1. Results and Analysis of Re-Identification Experiments

The cattle re-identification modeling experiments aim to address the challenge of varying appearance characteristics of the same cattle due to changes in shooting angles and lighting conditions. These variations often compromise the effectiveness of traditional object recognition and tracking methods. The re-identification model improves recognition accuracy and tracking stability across different times and environments by associating multiple appearance patterns of the same cattle in various scenes. This advancement is crucial for effective long-term monitoring and management of cattle.

Experiments were conducted using the Market-1501 dataset format to create a cattle re-identification dataset. The ResNet-50 model was employed for training cattle re-identification, and the trained weights were obtained. The model was trained on the Cattle ReID dataset with a batch size of 16 and 60 epochs, while other parameters were set to their default values.

**Figure 13:**
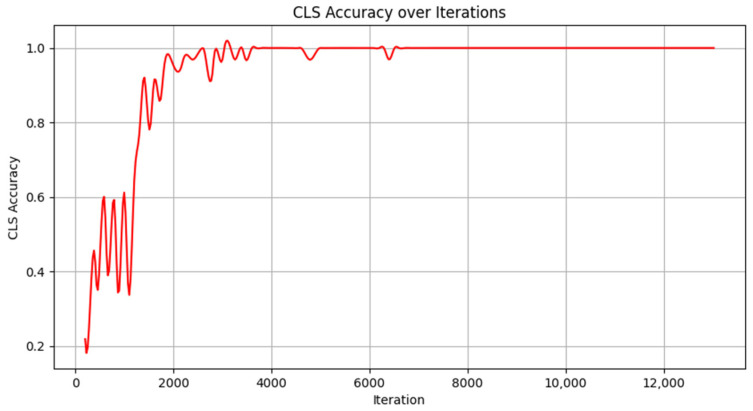
Variation curve of re-identification model accuracy.

Re-identification accuracy was monitored using TensorBoard during training. The vertical axis represents the model’s classification accuracy, with values closer to 1.0 indicating a stronger ability to extract features and reliably recognize the same cattle under varying conditions. The horizontal axis represents the number of training iterations. The results, shown in Figure 13, illustrate how classification accuracy trends with increasing training iterations. When the number of training iterations reaches 7.39 × 10^3^ and the iteration period is 32, the classification accuracy curve stabilizes, indicating model convergence with an accuracy of 100%. These results demonstrate that the re-identification model effectively extracts appearance features of different cattle after adequate training and can accurately distinguish between individual cattle.

#### 3.3.2. Comparison of Effects before and after Improvement of the Algorithm

To validate the performance of the multi-object tracking algorithm, 10 videos from different scenarios were used as test videos. The tracking experiment results for these 10 videos are shown in Table 6, the IoU ablation experiment results are shown in Table 7, and the performance comparison of the model before and after improvements is presented in Table 8.

According to the results in Table 6, variations in tracking accuracy metrics can be attributed to differences in video environments, such as background, light intensity, time of day, cattle density, and occlusion conditions. For instance, Video 04, filmed at 7:00 p.m., demonstrated lower tracking performance due to poor lighting and severe occlusion, compounded by the presence of a background wall. The low contrast between the cattle and the wall, exacerbated by the cattle’s darker fur color, made accurate identification and tracking challenging, resulting in a lower HOTA score. In contrast, Video 06, despite being recorded at night under poor lighting conditions, achieved the best tracking performance. This was due to the sparse distribution of cattle and minimal interference from shadows, which facilitated more accurate tracking.

To further demonstrate the effectiveness of DIoU in multi-object tracking algorithms, an ablation study was conducted on the cattle tracking dataset. The experiment compared the performance of different bounding box regression methods, including IoU, GIoU, BIoU, and DIoU, to evaluate their impact on cattle tracking in complex farming environments. The results, as shown in Table 7, indicate that DIoU performed the best in the multi-object tracking task for cattle in farm settings. DIoU excelled in the localization accuracy of detection boxes and data association consistency, effectively reducing identity switching and improving overall tracking accuracy. This makes it particularly suitable for addressing the challenges of cattle behavior monitoring in complex environments.

As shown in Table 8, while the improved YOLOv8n algorithm resulted in a decrease in tracking inference speed and accuracy compared to the original YOLOv8n model, it significantly enhanced other accuracy metrics, demonstrating its effectiveness. Additionally, after incorporating the improved BoTSORT algorithm, metrics such as HOTA, MOTP, MOTA, IDF1, and MTR showed improvement, and the number of identity switches (IDS) was reduced by four. This indicates that replacing the DIoU distance calculation with reclassified confidence detection frames effectively enhances tracking performance and minimizes identity switching issues.

**Figure 14:**
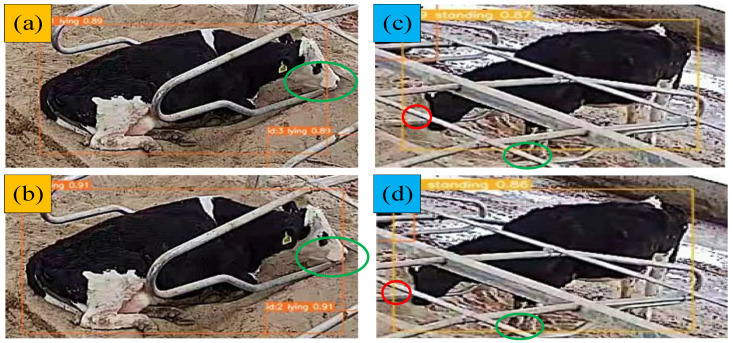
Comparison of the improved results of replacing DIoU, (**a**,**c**) denote the tracking results of the original algorithm, and (**b**,**d**) denote the tracking results of the improved algorithm. The green circle indicates the part of the target extending beyond the detection box, while the red circle indicates the detection box containing extra background information.

The tracking results of the improved BoTSORT algorithm are illustrated in Figure 14. In Figure 14a, the original algorithm’s tracking frame only partially captures the cattle’s mouth. In contrast, Figure 14b shows that the improved algorithm provides a more precise tracking frame that accurately encompasses the cattle’s mouth. Similarly, Figure 14c demonstrates that the original tracking frame includes more background, whereas Figure 14d shows that the improved tracking frame is closer to the cattle and includes less background. This indicates that the enhanced BoTSORT algorithm offers superior performance in accurately aligning the tracking frame with the cattle object.

**Figure 15:**
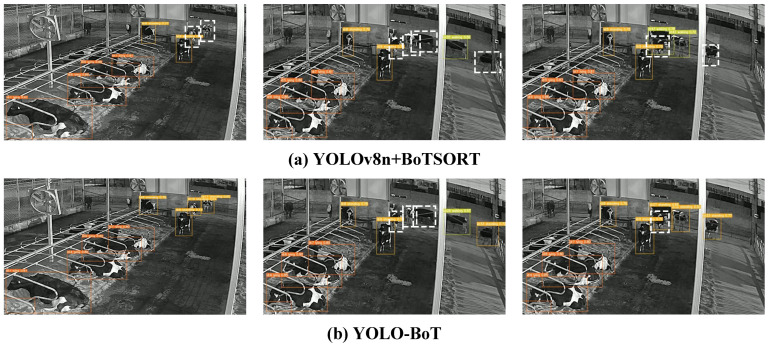
Comparison between before and after the tracking algorithm improvement at frame 50, frame 652, and frame 916, respectively. The white dotted line in the image indicates the untracked object.

Figure 15 compares the performance of the model before and after improvement using Video 01, which features low light, occlusion, and other disturbances. In Figure 15a, issues such as low light, mutual occlusion among cattle, and the color similarity of some cattle with the background result in difficulties in detection and tracking, leading to tracking failures. In contrast, Figure 15b shows that the improved model successfully tracks the corresponding objects despite these challenges. However, due to the darker background in the video, both the original and improved models miss some objects, as indicated by the white dashed lines in frames 652 and 916 of Figure 15a,b.

**Figure 16:**
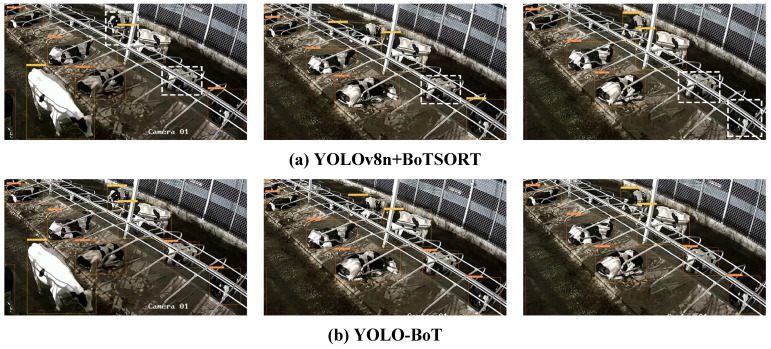
Comparison between before and after the tracking algorithm improvement at frame 22, frame 915, and frame 1504, respectively. The white dotted line in the image indicates the untracked object.

Video 09 was captured at night in a lit environment with glare and significant shading between cattle. Figure 16 shows the tracking results before and after model improvement. In Figure 16a, the original model struggles with object loss during tracking, as indicated by the white dashed boxes in frames 22, 915, and 1504. In contrast, Figure 16b demonstrates that the improved model effectively handles lighting and occlusion interference, accurately tracking each cattle and significantly reducing missed detections. However, due to the complex environment, some cattle with similar appearances lead to ID switching, such as cattle 79, 82, and 111 in Figure 16a and cattle 68 and 76 in Figure 16b. Overall, the improved model shows superior tracking performance in complex environments compared to the original model.

#### 3.3.3. Comparison of Different Multi-Object Tracking Algorithms

**Figure 17:**
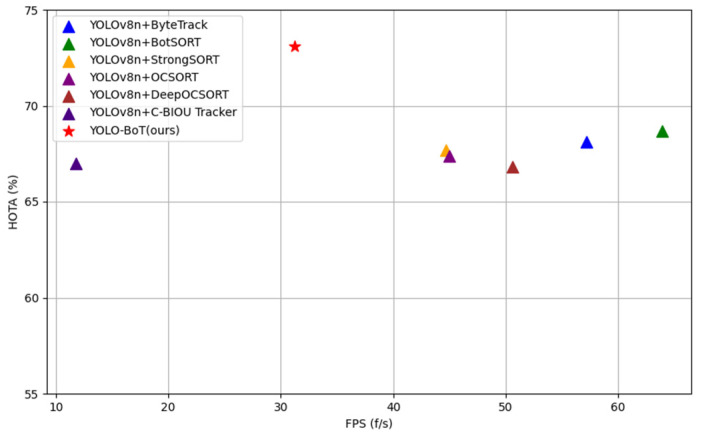
Performance comparison of tracking algorithms.

To validate the performance of the proposed cattle multi-object tracking methods, tests were conducted on the cattle multi-object tracking dataset. Six commonly used MOT tracking algorithms were selected for comparison. The results are summarized in Table 9 and illustrated in Figure 17.

As shown in Figure 17, the YOLO-BoT algorithm achieved the highest HOTA score, indicating superior tracking performance. According to Table 9, although the inference speed of the improved algorithm is relatively lower, its overall accuracy metrics are significantly higher, proving that the improved model delivers the best tracking performance. Comparing similar tracking algorithms, it can be observed that OCSORT and DeepOCSORT perform the worst in terms of tracking, with IDS values of 476 and 428, respectively. In contrast, the C-BIoU Tracker had the fewest IDS instances due to its enhanced bounding box overlap measurement strategy and more sophisticated object association and detection methods, which reduced the number of ID switches in complex scenarios. In terms of accuracy indicators such as HOTA, MOTA, MOTP, and IDF1, YOLO-BoT outperforms the others. Compared with the ORU mechanism in OCSORT, YOLO-BoT’s virtual trajectory update mechanism, which utilizes confidence score reallocation and DIoU distance calculation, effectively reduces false detections and improves tracking accuracy. Furthermore, the strategy of discarding low-confidence detection boxes further minimizes false positives and optimizes the data association process, thereby enhancing overall tracking performance. The algorithm also showed the best tracking success rate, reaching 72.6%, demonstrating its effectiveness in capturing real targets. Moreover, it had the lowest tracking failure rate, indicating fewer false alarms. The total IDS for the YOLO-BoT algorithm was 78, slightly higher than the 64 of the C-BIoU Tracker, but still significantly lower than other comparison algorithms, suggesting that YOLO-BoT exhibits fewer ID switches and higher performance stability across different environments. In summary, the proposed method is better suited for multi-object tracking of cattle in complex farming scenarios.

**Figure 18:**
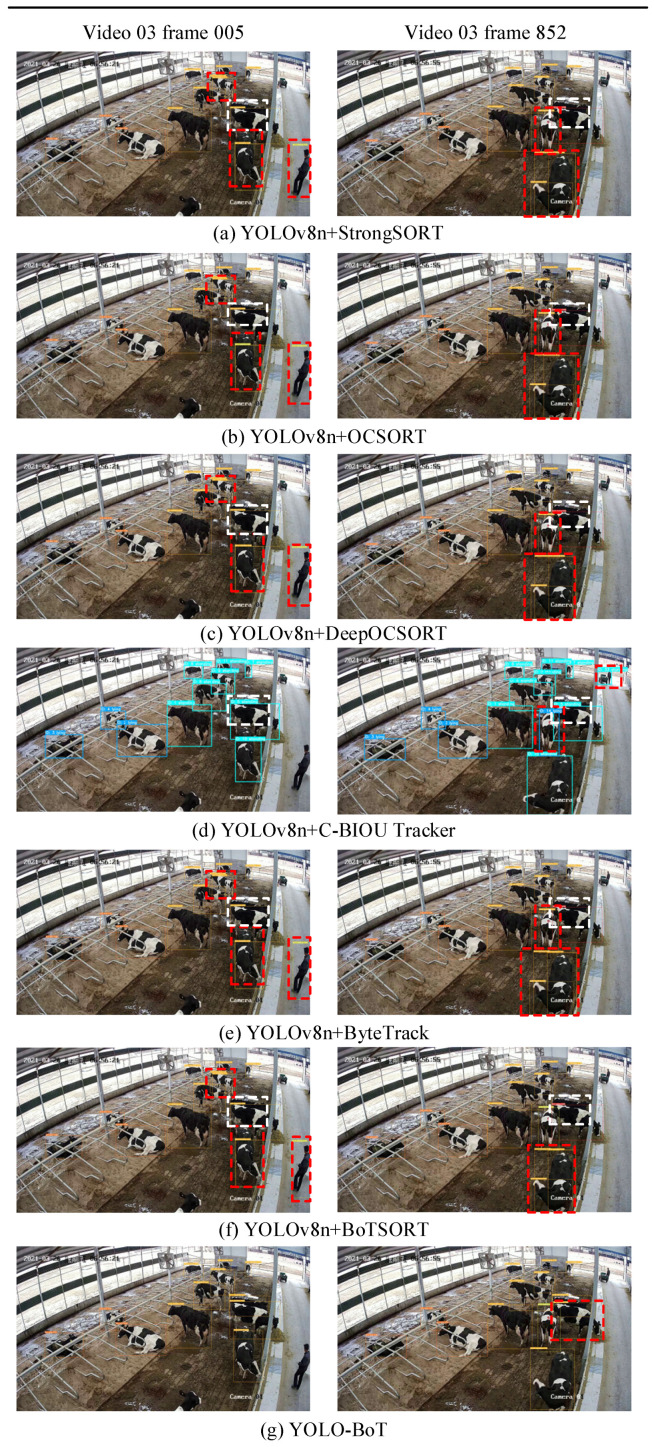
Tracking results for multiple tracking algorithms. White dashed lines in the image indicate untracked objects, while red dashed lines indicate incorrectly tracked objects. The time in the top-left corner of the image represents the capture time of the data.

The visualized tracking results of different algorithms for Video 03 are shown in Figure 18. This video features well-lit conditions with sparsely distributed cattle, but there is some interference from manual grass pushing and minor shading between some cattle. In Figure 18, it can be observed that all compared algorithms—BoTSORT, ByteTrack, StrongSORT, C-BIOU Tracker, OCSORT, and DeepOCSORT—exhibit missed detections during tracking, as indicated by the objects in the white dashed boxes in Figure 18a–f. In contrast, the method proposed in this study successfully tracked all objects without missing any, demonstrating effective detection and tracking despite the presence of partial dense occlusion. Regarding misdetections and repeated detections, five tracking algorithms encountered issues such as mistaking grass pushers for walking cattle and duplicating detections of the same cattle (objects in the blue dashed boxes in Figure 18a–c,e,f). In Figure 18d, distant humans and vehicles were mistakenly identified as standing cattle. However, the improved YOLOv8n algorithm integrated with BoTSORT, proposed in this study, did not suffer from duplicate detections. Nevertheless, there were instances where feeding behavior was misclassified as standing behavior. For example, in frame 852 of Video 03, the cattle with ID 2 (Figure 18g) exhibited a false detection issue. The model struggled to distinguish between feeding and standing behavior due to the shooting angle, which made the cattle’s lowered head appear parallel to the grass, leading the model to incorrectly classify the behavior.

In summary, this study demonstrates that by enhancing the YOLOv8n algorithm and integrating it with the BoTSORT algorithm, stable tracking performance can be achieved even in complex environments with dense occlusion. Compared to other multi-object tracking algorithms, the proposed method excels in tracking performance across diverse and challenging scenes. This advancement provides robust technical support for accurate cattle management, effectively enabling reliable multi-object tracking under various conditions and lighting environments.

#### 3.3.4. Analysis of the Duration of Long-Term Behavior of Cattle

Statistical data on cattle behavioral patterns within a specific time period can be obtained by analyzing the duration of each cattle’s behaviors. In this study, a 10 min video recorded between 12:24 PM and 12:34 PM was used to analyze the behavioral durations of both the herd and individual cattle [22].

**Figure 19:**
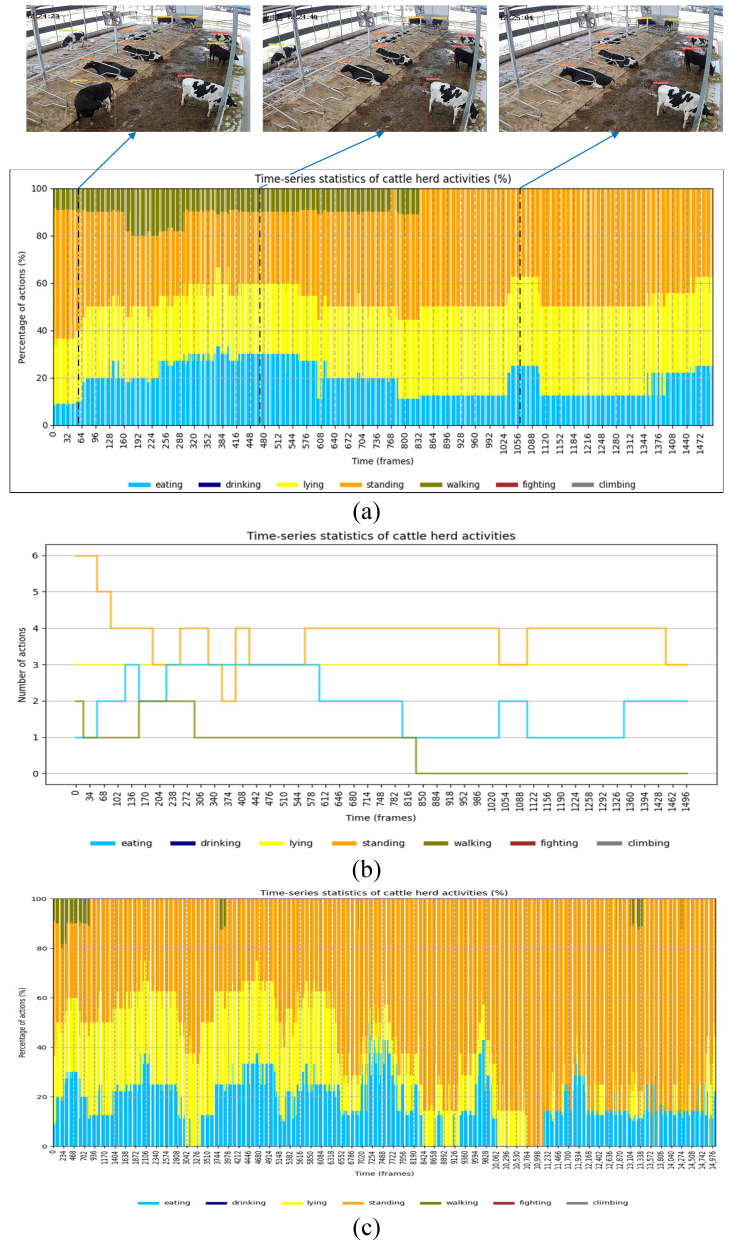
Behavioral duration data from the herd are displayed in one minute, focusing on the incidence of the behavior (**a**) and the number of individual cattle (**b**). Expanded to the entire 10 min video (**c**) to fully demonstrate behavioral changes in the herd over time.

Figure 19 presents the results of the overall herd behavior analysis, depicted in two formats: behavior incidence (Figure 19a) and the number of individuals exhibiting each behavior (Figure 19b). For example, the monitoring data reveal that approximately 50% of the cattle were standing, 20% were feeding, 20% were lying down, and about 10% were walking. To enhance the analysis, it is advisable to use a 10 min video, as illustrated in Figure 19c. An extended video duration provides a more comprehensive view of behavioral changes and trends within the herd.

**Figure 20:**
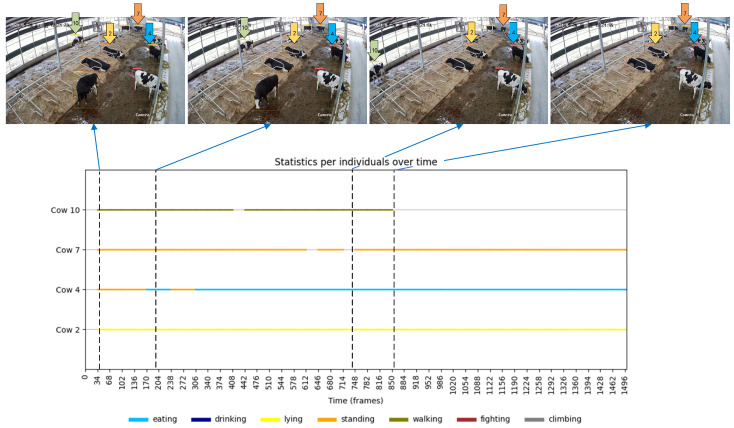
Time series statistics for each cattle over a one-minute period. Four cattle with both active and quiet behavior were specifically chosen to demonstrate these variations. The numbers 2, 4, 7, and 10 indicate the scaling of the selected cattle IDS assigned by the model in the initial frame.

In addition to the overall herd behavior analysis, detailed data on the individual cattle’s IDS can be obtained using a tracker, which depends on the stability of the tracking system and the accuracy of behavior detection. Figure 20 illustrates the analysis of four selected cattle, each exhibiting typical behavioral changes across different activity ranges. Specifically, the cattle with ID 2 remained lying down throughout the monitoring period; the cattle with ID 4 transitioned from standing to feeding; the cattle with ID 7 encountered an object loss during tracking, as shown by the missing detection frame in Figure 20, frame 746; and the cattle with ID 10 gradually exited the camera frame, leading to a subsequent loss of detection. The information presented in the figure highlights the model’s capability to analyze the behavioral duration of individual cattle, offering insights into their behavioral changes over a specific time period. It is important to note that the accuracy of object detection and the stability of the tracker are critical factors in obtaining reliable behavioral data.

### 3.4. Discussion

The multi-object tracking method based on a single surveillance camera proposed in this study demonstrates higher accuracy under real-time tracking conditions compared to existing commonly used algorithms. This is achieved by effectively mitigating the effects of illumination variations, object scale inconsistency, occlusion, and frequent switching of identities on tracking accuracy in complex environments. The method not only tracks multiple objects stably, but also provides in-depth statistical analysis of the behavioral changes of groups and individual cattle, offering strong technical support for the management and monitoring of modern animal husbandry.

Although the model showed significant improvements in accuracy and related metrics, experimental results still reveal certain issues. As illustrated in Figure 15, Figure 16 and Figure 18, the improved model mitigates the impact of complex environments on tracking precision to some extent, but false negatives and false positives are yet to be fully resolved. These issues may be related to the complexity of the model itself. While the current YOLOv8n model operates at a high speed, its limited parameter capacity and feature extraction capability may pose challenges in handling complex scenarios. Future work could consider adopting larger versions of the model, such as YOLOv8s or YOLOv8m, to enhance feature extraction and further improve detection accuracy and robustness. In addition, incorporating synthetic data generated from computer simulations could help expand the diversity and scope of the training dataset. For example, following the approach suggested by Foszner [35], it would be feasible to create synthetic datasets simulating cattle behavior under various conditions, such as different lighting, backgrounds, and occlusion scenarios. These synthetic data would effectively complement the real-world dataset by covering complex scenes that are otherwise underrepresented, thereby improving the model’s generalization ability and reducing missed and false detections in challenging environments. On the other hand, state-of-the-art tracking algorithms such as MotionTrack [36], OmniTracker [37], and NCSiam [38] offer valuable insights for our research in occlusion handling and complex scenarios. Specifically, MotionTrack leverages its interaction module to effectively capture dynamic relationships between densely packed targets, making it well-suited for managing interactions in crowded cattle herds. OmniTracker employs a multi-task feature fusion strategy, maintaining high tracking accuracy under complex occlusions and deformations. NCSiam utilizes a multi-level similarity optimization strategy, demonstrating strong target discrimination in cluttered backgrounds. These methods provide valuable references for improving multi-object tracking performance and can help further enhance the robustness and monitoring effectiveness of our system in complex environments.

Additionally, as shown by the data collection locations of the surveillance videos in Figure 1, the fixed camera’s location limits its surveillance range, failing to cover the entire barn area and resulting in a blind spot in the surveillance data. For instance, the cattle with ID 10 in Figure 20 could not be tracked further after stepping out of the monitoring range, making it impossible to accurately monitor the cattle once it entered the blind zone.

To address the challenges of occlusion and blind spots in the current monitoring system within complex cattle barns, future research will focus on exploring multi-agent collaborative multi-object tracking technologies. Specifically, a combination of fixed cameras and mobile cameras (such as patrol robots) can be employed for joint monitoring. Fixed cameras will cover the primary areas, enabling real-time monitoring of large-scale cattle activities and providing foundational data collection. Meanwhile, mobile patrol robots can complement the monitoring in areas not covered by the fixed cameras, dynamically adjusting their positions and angles to focus on key targets or specific regions, offering more detailed behavioral data. This multi-agent collaboration enables comprehensive, multi-layered monitoring, significantly expanding system coverage and improving monitoring accuracy, ensuring that cattle behavior can be thoroughly captured and analyzed under various barn conditions.

Additionally, future research can leverage multi-camera tracking technologies to further enhance monitoring effectiveness. For example, by using multi-view data fusion methods, information from multiple cameras can be integrated to eliminate the limitations of single-camera viewpoints, resulting in more comprehensive and accurate target tracking outcomes. Methods proposed by Zhou et al. [39], such as normalized epipolar distance calculation and propagated non-parametric clustering (PDNC), can effectively enhance cross-camera data association capabilities. These methods could be applied to improve the robustness of cattle behavior identification and tracking in complex cattle barn environments, providing vital support for continuous cross-region tracking.

Future research will aim to explore various emerging technologies and methodologies to continually improve the accuracy and stability of cattle behavior monitoring, optimize system adaptability and response speed, and achieve more efficient resource utilization and intelligent management. These advancements will offer sustainable technological support for modern livestock farming.

## 4. Conclusions

In this study, we developed a multi-object behavior recognition and tracking model for cattle based on enhanced YOLOv8 and BoTSORT algorithms. This model enables stable tracking of cattle on farms and facilitates the statistical analysis of long-term behavioral changes. The main conclusions are as follows:

1. The improved YOLOv8n algorithm is designed to address issues such as uneven cattle distribution, significant changes in occlusion and object scale, and low tracking accuracy due to frequent identity switches. To tackle uneven distribution and object scale variations, a dynamic convolution (DyConv) module is integrated into the model’s backbone. The C2f-iRMB structure is utilized in the neck part to enhance feature characterization and reduce computational complexity. Additionally, the downsampling ADown module is introduced to expand the sensory field for improved feature fusion, while the dynamic object detection head (DyHead) module is replaced to better integrate contextual information and enhance multi-scale object detection performance.
2. The proposed enhanced BoTSORT algorithm, which reclassifies high-confidence detection boxes and eliminates low-scoring ones, improves matching accuracy while reducing false alarms and identity switching issues. Additionally, the introduction of DIoU distance calculation further refines matching accuracy. To address inaccurate trajectory predictions caused by object loss, a virtual trajectory update mechanism is employed to minimize the accumulation of prediction errors. Once the activity trajectories of the cattle are obtained, statistical analysis and examination of their long-term behavioral changes are conducted.
3. The experimental results demonstrate that the proposed model achieves a mean average precision (mAP) of 91.7% on the cattle object detection dataset. It shows improvements of 4.4% in precision (P) and 1% in recall (R) over the original algorithm. Regarding tracking performance, the model exhibits a 4.4% improvement in HOTA, a 7% increase in MOTA, a 1.7% rise in MOTP, a 4.3% enhancement in IDF1, and a 30.9% reduction in IDS. The system operates at a frame rate of 31.2 frames per second (FPS). These results indicate that the proposed method effectively handles multi-object tracking of cattle in complex environments and offers valuable technical support for analyzing long-term behaviors and enabling non-contact automatic monitoring.

## Figures and Tables

**Table 1:** Cattle object tracking dataset.

No.	Weather	Period	Sparse	Dense	Interference Factors
01	Cloudy	Sunrise		√	Wall shading, low light, cattle occlusion (medium)
02	Sunny	Sunrise	√		Moderate light, artificial push grass disturbance, cattle occlusion (medium)
03	Sunny	Morning	√		Manual push grass disturbance, cattle occlusion (light)
04	Cloudy	Evening	√		Walls, behavioral changes, and cattle occlusion (Medium)
05	Sunny	Night (with lights)	√		Strong light, cattle occlusion (light)
06	Sunny	Night (no lights)	√		Low light, camera wobbles slightly, cattle occlusion (light)
07	Sunny	Evening		√	Varying behavior, cattle occlusion (heavy)
08	Cloudy	Morning		√	Varying behavior, cattle occlusion (heavy)
09	Sunny	Night (with lights)		√	Strong light, cattle occlusion (heavy)
10	Cloudy	Night (no lights)		√	Low light, cattle occlusion (heavy)

**Table 2:** Results of 5-fold cross-validation experiment.

Fold Number	P/%	R/%	mAP/%	F1/%
Fold 1	87.0	92.0	93.1	89.4
Fold 2	89.6	88.9	93.4	89.2
Fold 3	92.0	87.5	92.0	89.7
Fold 4	91.2	86.9	92.1	89.0
Fold 5	90.6	86.9	90.9	88.7
Average	90.1	88.4	92.3	89.2
Standard deviation	1.7	1.9	0.9	0.3

**Table 3:** Comparison of the experimental results.

Dataset	P/%	R/%	mAP/%	F1/%
Fold 2	89.6	88.9	93.4	89.2
Test Set	90.9	84.7	91.7	87.7

**Table 4:** Experimental results of different models.

Models	P/%	R/%	mAP/%	FPS	Params/ 10^6^	GFLOPs/ 10^9^	Model Size/MB
YOLOv3-tiny	88.8	82.1	89.9	123.4	12.1	18.9	24.4
YOLOv5n	87.0	83.0	90.0	74.6	2.5	7.1	5.3
YOLOv6n	88.1	85.4	90.7	83.3	4.2	11.8	8.7
YOLOv7-tiny	88.6	87.5	91.3	27.8	6.0	13.3	12.3
RTDETR-r18	90.1	88.4	90.0	36.2	19.8	57.0	40.5
YOLOv8n	86.5	83.7	90.2	76.9	3.0	8.1	6.2
YOLOv9t	82.8	84.8	90.7	35.3	1.8	7.1	4.5
YOLOv10n	87.0	79.3	88.3	48.3	2.7	8.2	5.5
Improved YOLOv8n	90.9	84.7	91.7	55.1	3.4	7.5	7.2

**Table 5:** Ablation experiment results.

Models	mAP	Ap						
		Feeding	Drinking	Standing	Lying	Walking	Climbing	Fighting
YOLOv8n	90.2	85.8	94.6	83.3	92.4	82.2	97.0	96.2
YOLOv8n + C2f-DyConv	90.5	83.8	94.5	83.3	90.2	83.3	99.5	99.5
YOLOv8n + C2f-iRMB	90.5	93.4	94.1	84.5	90.0	84.3	97.5	99.5
YOLOv8n + DyHead	91.3	85.6	96.6	85.0	89.3	83.9	99.4	99.5
YOLOv8n + Adown	91.2	86.5	94.4	85.3	90.4	85.4	97.1	99.5
Improved YOLOv8n	91.7	87.4	95.5	85.1	88.4	86.8	99.1	99.5

**Table 6:** Experimental results on the cattle-only tracking dataset. Metrics marked with ↑ indicate that higher values represent better performance, while those marked with ↓ indicate that lower values are better.

No.	HOTA/% (↑)	MOTA/% (↑)	MOTP/% (↑)	IDF1/% (↑)	MTR/% (↑)	MLR/% (↓)	IDS (↓)	FPS(f/s) (↑)
01	66.4	69.2	85.2	79.5	43.5	21.7	10	30.8
02	83.4	96.5	84.9	97.9	90	0	1	32.3
03	80.9	85.5	89.5	92.2	85.7	14.3	0	31.1
04	47.6	53.1	74.6	69.4	44.4	22.2	0	31.8
05	81.7	88.7	87.3	94.7	85.7	0	0	31.4
06	83.6	96.5	84.9	98.2	90	0	0	29.9
07	74.5	84.3	83.1	88.3	69.0	3.4	44	33.4
08	71.1	76.9	83.8	86.1	72.7	13.6	2	29.4
09	70.9	77.6	84.6	84.3	70.8	25	18	30.7
10	70.7	75.3	85.8	83.9	73.7	26.3	3	30.8
Overall	73.1	80.4	84.4	87.5	72.6	12.7	78	31.2

**Table 7:** Ablation experiment results. The ▲ symbol represents the use of the improved YOLOv8 model with redefined confidence threshold selection and the introduction of the virtual trajectory update mechanism. IoU, GIoU, BIoU, and DIoU correspond to different bounding box regression methods. ↑ indicates higher is better, while ↓ indicates lower is better.

Models	HOTA/% (↑)	MOTA/% (↑)	MOTP/% (↑)	IDF1/% (↑)	MTR/% (↑)	MLR/% (↓)	IDS (↓)	FPS(f/s) (↑)
▲ + IoU	72.9	80.1	82.4	87.2	72.0	12.2	82	33.7
▲ + BIoU	72.9	80.1	82.5	86.9	72.5	11.9	82	33.2
▲ + GIoU	73.0	80.2	83.1	87.5	72.2	12.7	79	31.7
▲ + DIoU	73.1	80.4	84.4	87.5	72.6	12.7	78	31.2

**Table 8:** Tracking results before and after improvement of algorithm. ↑ indicates higher is better, while ↓ indicates lower is better.

Models	HOTA/% (↑)	MOTA/% (↑)	MOTP/% (↑)	IDF1/% (↑)	MTR/% (↑)	MLR/% (↓)	IDS (↓)	FPS(f/s) (↑)
YOLOv8n + BoTSORT	68.7	73.4	82.7	83.2	66.3	16.8	113	63.9
Improved YOLOv8n + BoTSORT	72.9	80.1	82.4	87.2	72.0	12.2	82	33.7
YOLO-BoT	73.1	80.4	84.4	87.5	72.6	12.7	78	31.2

**Table 9:** Multi-object tracking results. ↑ indicates higher is better, while ↓ indicates lower is better.

Models	HOTA/% (↑)	MOTA/% (↑)	MOTP/% (↑)	IDF1/% (↑)	MTR/% (↑)	MLR/% (↓)	IDS (↓)	FPS(f/s) (↑)
YOLOv8n + ByteTrack	68.1	72.2	82.6	81.8	65.9	20	104	57.2
YOLOv8n + BoTSORT	68.7	73.4	82.7	83.2	66.3	16.8	113	63.9
YOLOv8n + StrongSORT	67.7	72.2	82.7	80.8	65.5	21.8	258	44.7
YOLOv8n + OCSORT	67.4	72.2	82.6	80.1	65.5	19.7	476	45.0
YOLOv8n + DeepOCSORT	66.8	72.2	82.6	79.1	65.5	19.7	428	50.6
YOLOv8n + C-BIoU Tracker	67.0	70.5	82.8	81.9	64.8	22.8	64	11.8
YOLO-BoT	73.1	80.4	84.4	87.5	72.6	12.7	78	31.2

## Data Availability

The data presented in this study are available on request from the corresponding author. The data are not publicly available due to ongoing study.
